# Pre-ingestive selection capacity and endoscopic analysis in the sympatric bivalves *Mulinia edulis* and *Mytilus chilensis* exposed to diets containing toxic and non-toxic dinoflagellates

**DOI:** 10.1371/journal.pone.0193370

**Published:** 2018-02-23

**Authors:** Jorge M. Navarro, John Widdows, Oscar R. Chaparro, Alejandro Ortíz, Carla Mellado, Paola A. Villanueva

**Affiliations:** 1 Instituto de Ciencias Marinas y Limnológicas, Universidad Austral de Chile, Valdivia, Chile; 2 Centro FONDAP de Investigación en Dinámica de Ecosistemas Marinos de Altas Latitudes (IDEAL), Valdivia, Chile; 3 Plymouth Marine Laboratory, Prospect Place, West Hoe, Plymouth, England; Museum National d’Histoire Naturelle, FRANCE

## Abstract

This study investigates the effects of toxic and non-toxic dinoflatellates on two sympatric bivalves, the clam *Mulinia edulis* and the mussel *Mytilus chilensis*. Groups of bivalves were fed one of three diets: (i) the toxic paralytic shellfish producing (PSP) *Alexandrium catenella* + *Isochrysis galbana*; (ii) the non-toxic *Alexandrium affine* + *Isochrysis galbana* and (iii) the control diet of *Isochrysis galbana*. Several physiological traits were measured, such as, clearance rate, pre-ingestive selection efficiency and particle transport velocity in the gill. The clearance rates of both *M*. *chilensis* and *M*. *edulis* showed a significant reduction when fed a mixed toxic diet of 50% *Alexandrium catenella*: 50% *Isochrysis galbana*. Similarly, when both species of bivalves were fed with the non-toxic diet (50% *A*. *affine*: 50% *I*. *galbana*), clearance rate was significantly lower compared with a diet of 100% *I*. *galbana*. Under all the experimental diets, *M*. *chilensis* showed higher clearance rate values, slightly more than double that of *M*. *edulis*. *M*. *edulis* and *M*. *chilensis* have the ability to select particles at the pre-ingestive level, thus eliminating a larger proportion of the toxic dinoflagellate *A*. *catenella* as well as the non-toxic *A*. *affine* in the form of pseudofaeces. Higher values of selection efficiency were registered in *M*. *edulis* than in *M*. *chilensis* when exposed to the toxic diet. Similar results were observed when these two species were exposed to the diet containing the non-toxic dinoflagellate, explained by the fact that the infaunal *Mulinia edulis* is adapted to dealing with larger particle sizes and higher particle densities (Navarro et al., 1993). The lower transport particle velocity observed in the present work for both species, is related to the reduced clearance rate, the higher particle concentration, and the presence of larger, toxic dinoflagellates. In addition, the species differ in their feeding responses to diets, with and without *A*. *catenella* or *A*. *affine*, largely reflecting their adaptations to different environmental conditions. The results suggest that the presence of a dinoflagellate bloom, whether toxic or non-toxic spp in Yaldad Bay, is likely to have a greater impact on the *Mytilus chilensis* than the infaunal *Mulinia edulis*, based on the combined effects on clearance rate, selection efficiency and particle transport velocity.

## Introduction

Suspension feeding bivalves live in environments characterized by large fluctuations in the quantity and quality of the suspended particulate matter. They are exposed to different particle sizes, with variable nutritional content that is sometimes mixed with toxic algal cells. The functional response of filter-feeding organisms to fluctuations in the quantity and quality of suspended particulate matter have been studied by many authors [1–5]. Filter-feeding bivalves can compensate for food quality reductions through physiological mechanisms that improve the energy gain from environments characterised by large fluctuations of the suspended particulate matter. Filter-feeding bivalves can compensate for the “dilution” of desirable particles (i.e. nutritive and/or non-toxic) by undesirable particles (i.e. inorganic and/or toxic) contained in the food supply, through preferential ingestion of desirable particles and selective rejection of undesirable particles via the pseudofaeces [1,3,6–9]. The exact mechanisms used by suspension-feeding bivalves for determining which particles are ingested or rejected as pseudofaeces remain unknown. Selection may involve a physico-chemosensory response by the feeding organs. Particle selection in bivalves could be related to the specific chemical interaction between lectins in the mucus of pallial organs and carbohydrates present on the surfaces of the suspended particles [10,11]. This selective feeding behaviour has been observed in bivalves as a response to the presence of toxic dinoflagellates [1,9].

Harmful algal blooms (HABs) leading to paralytic shellfish poisoning (PSP) events have increased worldwide [12,13] and dinoflagellates of the genus *Alexandrium* are the primary source of the paralytic toxin. The impacts of this genus result in serious economic losses for the shellfish industry and have a negative impact on public health. Numerous authors have studied the impact of HABs on marine filter feeders [14–18] and different physiological and behavioural effects have been described in bivalve species exposed to diets containing PSP [1,19–22]. The results suggest that the different species of filter feeding bivalves have different responses. Some mussels (*Modiolus modiolus*, *Mytilus edulis*, *Perna caniliculus*, *Choromytilus chorus*) appear to be highly unresponsive when feeding on toxic dinoflagellates [14,23–25], but *Mytilus chilensis* shows an initial reduction in filtration activity during the first hours of exposure to *A*. *catenella*, but returning to normal within 48 h [26]. In contrast, the feeding activity and the growth rate of juvenile and adult oysters and clams were reduced when exposed to algal cells containing PSP [22,27–29]. Similarly, the oyster *Crassostrea gigas* [30] and the clam *Ruditapes philippinarum* [31], significantly reduced filtration activity when exposed to the toxic dinoflagellate *Protogonyaulax tamarense* strain GT429 (= *Alexandrium tamarense*). The reduction in the feeding rate affects the energy intake of these organisms, thus reducing the energy available for growth and reproduction, which can negatively impact species fitness [22]. Shumway and Cucci [14] found that most specimens of the clam *Mya arenaria* showed withdrawn or partially withdrawn siphons, which remained retracted for extended periods and this was associated with a highly reduced clearance rate in the presence of *Protogonyaulax tamaresis* (strain GT429). These authors also found that *Mya arenaria* has the capacity, at the pre-ingestive level, to select non-toxic cells for ingestion and toxic dinoflagellate cells for rejection via the pseudofaeces. These authors concluded that the responses observed are species-specific and depend on the geographical origin of the organisms.

Blooms of the dinoflagellate *Alexandrium catenella* occur frequently in the most southerly administrative regions of Chile, and in the last decade the phenomenon has extended northward, reaching Chiloé Island, which is Chile’s principal bivalve cultivation area (42° 38’ S; 73° 65’ W). In early 2016, *A*. *catenella* blooms extended hundreds of kilometres northward to the Valdivian coast (39° 87’ S; 73° 40’ W). The occurrence of toxic dinoflagellate blooms in Southern Chile has a negative impact on local bivalve populations as well as to aquaculture activities, leading to a temporary shutdown of harvesting activities. Understanding how to reduce HABs impacts on bivalves is therefore important in the management of these areas.

The main aim of this study was to evaluate the effects of toxic and non-toxic dinoflagellates on the feeding behaviour of the mussel *Mytilus chilensis* and the clam *Mulinia edulis*, two common and important sympatric bivalves inhabiting the fjords of Southern Chile, where HABs have increased in frequency and intensity. For this purpose the following measurements were carried out:

i) feeding rate, ii) capacity to sort toxic and non-toxic dinoflagellate cells (two species of similar size and shape) at the pre-ingestive level, and iii) endoscopic analyses of particle transport on the gills.

## Materials and methods

### Experimental animals

Adult specimens (ca. 5.5 cm shell length) of the mussel *Mytilus chilensis* were collected at the same time from culture rafts and the clam *Mulinia edulis* from tidal flats of the Yaldad Bay, Chiloé (43° 08’ S, 73° 44’ W). Both species with similar length (ca. 5.5 cm) were collected at the same time during autumn season and transported to the laboratory, acclimatized for two weeks at 14 ° C, 30 psu, and fed continuously with the microalgae *Isochrysis galbana* (ca. 1.0 mg L^-1^). No specific permissions were required for this location. In Chile there is free access to the coast with the public coastal footpath around nearly the whole country. Only for the case of protected areas and National Parks it is necessary to ask special permission. Our field studies do not involve endangered or protected species.

### Experimental design and diet preparation

Monoclonal cultures of the toxic dinoflagellate *Alexandrium catenella* (strain ACC02) and the non-toxic dinoflagellate *Alexandrium affine* and haptophyte *Isochrysis galbana* were used as food in a series of feeding experiments. In all cases, we used the exponential growth phase of the microalgae. In order to generate different food concentrations and levels of toxicity, three experimental diets were prepared (10, 20 and 50 mg L^-1^), all containing 50% of *A*. *catenella* and 50% of *I*. *galbana* (based on mg L^-1^ but monitored in terms of cell concentration). The experiments were carried out using ten experimental aquaria (one bivalve per aquarium); five containing bivalves exposed to toxic diets (*A*. *catenella* 50% + *I*. *galbana* 50%) and five fed the control diet (*I*. *galbana* 100%). All experiments to determine clearance rate (CR) and pre-ingestive selection efficiency were carried out on three occasions.

The toxin concentration of *A*. *catenella* cells (strain ACC02) was 10.3 ± 0.9 fmoles STX eq. cell^-1^, according to measurements made in a parallel study [32]. Experimental and control diets had the same weight (mg L^-1^) at each different concentration. One separate experiment (repeated three times) was carried out using a diet free of PSP toxins at a single concentration of 10 mg L-1, containing 50% non-toxic *A*. *affine* and 50% *I*. *galbana*. Ten bivalves were exposed to toxic free dinoflagellates (*A*. *affine* 50% + *I*. *galbana* 50%) and ten fed the control diet (*I*. *galbana* 100%). Unfortunately, all subsequent attempts at cultivating *A*. *affine* from various different source cultures failed. To verify the complete absence of PSP toxins in *A*. *affine*, samples were sent to the Laboratorio de Toxinas Marinas at Universidad de Chile for quantification of their toxic content, electrophysiologically [33], which showed them to be non-toxic [26].

### Physiological measurements and endoscopy

#### Clearance rate

Clearance rate measurements were performed on individual specimens exposed to different diets prepared with *A*. *catenella*, *A*. *affine*, and with the control diet containing *I*. *galbana*. Food particle concentrations were monitored with an Elzone 180XY particle counter equipped with a 120 μm aperture counting tube. An aquarium without animals was included as a control, to allow for possible cellular sedimentation. Homogenization of experimental medium was maintained by air diffusers, and the experimental specimens were left undisturbed for 1 hour, to allow them to open their valves and begin filtering, before initiating CR measurements. The experiments were performed for a period of 6 hours, with measurements every 30 minutes, and clearance rate (L h^-1^) was determined on single individuals using the Coughlan method [34]. The consumed cells were replaced every 30 minutes, following each measurement, returning the algal concentrations to their initial food concentrations.

### Pre-ingestive selection efficiency

Experimental mussels and clams were exposed to three food concentrations of toxic diets (equal mixtures of *A*. *catenella* + *I*. *galbana*; 10, 20 and 50 mg L^-1^), and to one non-toxic concentration (10 mg L^-1^) of equal mixtures of *A*. *affine* + *I*. *galbana*. The different experimental diets were over the threshold for pseudofaeces production [35]. Pseudofaeces were identified as particles wrapped in mucus that are rejected from the inhalant siphon and deposited as a separate pile from the faeces. At the end of each experiment, pseudofaeces were collected and disintegrated by one minute using a slow speed vortex for one minute. Pseudofaeces from each treatment were resuspended in filtered seawater and counted with an inverted microscope (Utermöhl method) for determining *Alexandrium*/*Isochrysis* cell proportions. Selection efficiency was calculated according to the formula given by Bayne and Hawkins [35]:

$$SE=1-(\frac{p}{f})$$

Where: f and p represent the proportion of *Alexandrium/Isochrysis* in the food and pseudofaeces, respectively. SE = 0 means that there is no selection (f = p) and SE = 1 means complete selection and ingestion of only *Isochrysis* cells.

### Particle transport on the gills

Endoscopic examinations were carried out using an Olympus OTV-S4 system, a rigid optic of 1.7 mm diameter, and a xenon light source. The system was connected to a video camera, a monitor, and a video recorder to account for movement and estimate the transport velocity of food particles. A series of six experiments were carried out, each using three different individuals (replicates) of each species (approximately 5.5 cm shell length). These were exposed to a food concentration of ca. 2.0 mg L^-1^, consisting of a toxic diet of *A*. *catenella* + *I*. *galbana* (50 + 50% based on mass, mg L^-1^) and a control diet of *I*. *galbana* (100%). During the endoscopic observations, the experimental individuals were fixed to plastic panels to keep them in a suitable position for the introduction of the endoscope tip at an angle of approximately 45°. In order to introduce the endoscope into the pallial cavity, the valves were perforated on one side and the animals acclimated for 2 days to allow recovery. Since the particle transport of these two species occur only in the ventral groove of the gills [32], velocity was calculated by following a food particle along the ventral groove and counting the filaments crossed by the particle in a set amount of time. Gill filaments and the space between them were measured on 5 gill pieces using an ocular graduated microscope. Transport velocity was then calculated using the relationship between the time and distance travelled by the particles.

### Statistical analyses

A two-way ANOVA was used to estimate the effects of diet and food concentration on the clearance rates of individuals fed with the toxic diet and also to estimate the effects of diet and species on the clearance rate of individuals fed with *A*. *affine*. Tukey HSD was employed as a posteriori test on each factor that showed significant differences [36]. A two-way blocked ANOVA was conducted to analyse effects of diet (control and toxic) and species (*M*. *edulis* and *M*. *chilensis*) on particle transport velocity (experiments as random factor). Normality and homoscedasticity of the data were tested using Kolmogorov—Smirnov and Bartlett tests, respectively. All analyses were performed with RStudio, version 3.3.1, using the GAD library.

## Results

### Clearance rate studies

#### Exposure to toxic dinoflagellate *Alexandrium catenella*

**Fig 1:**
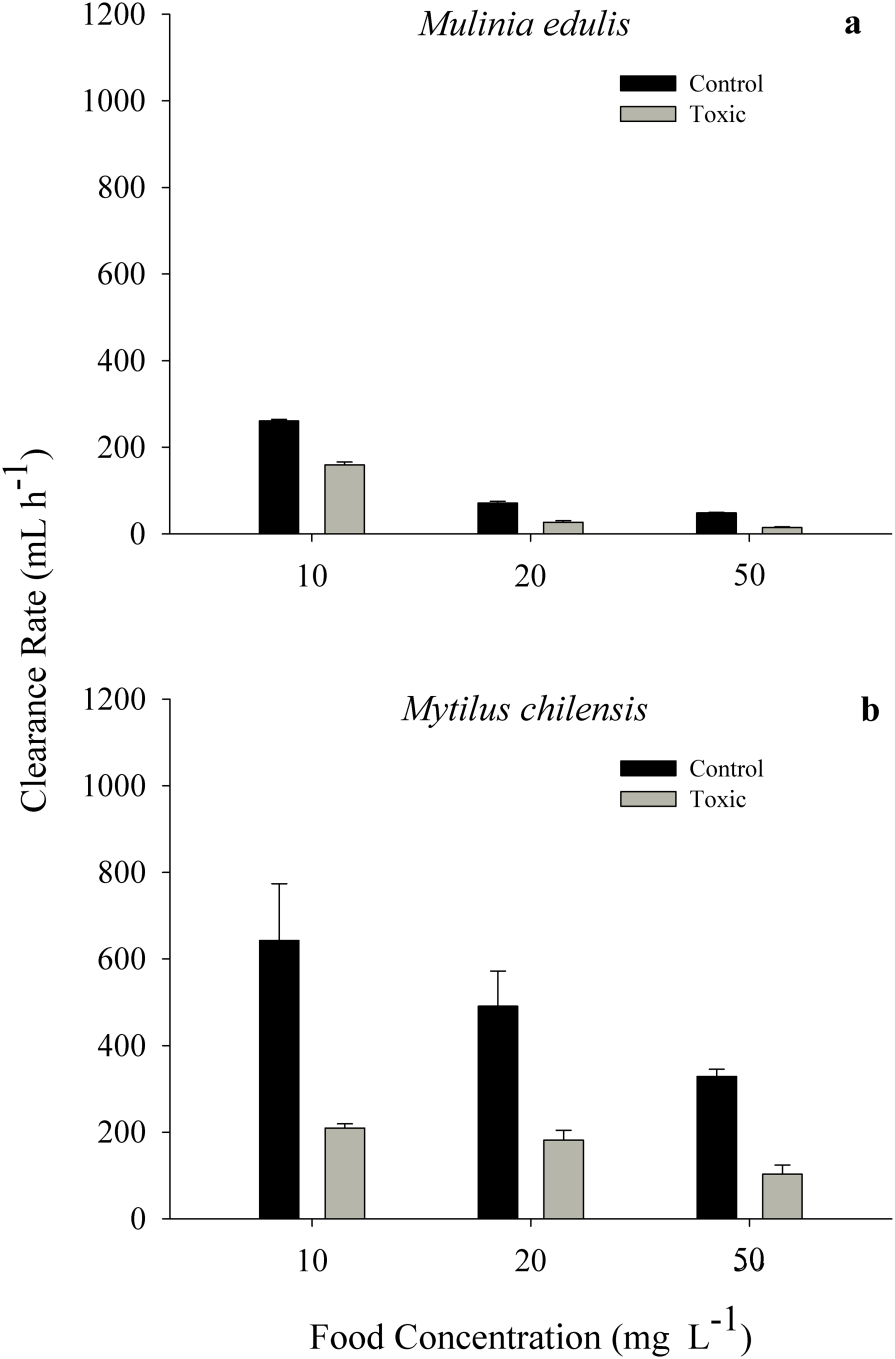
Clearance rate of *Mulinia edulis* (a) and *Mytilus chilensis* (b) exposed to different food concentrations (10, 20, 50 mg L^-1^) of a toxic diet (50% *Alexandrium catenella*: 50% *Isochrysis galbana*) and a control diet (100% *Isochrysis galbana*). Values represent means ± SE.

Significantly lower clearance rates (CR) were recorded when the clam *Mulinia edulis* and the mussel *Mytilus chilensis* were exposed to the toxic diet and when food concentration increased from 10 to 50 mg L^-1^. The two-way ANOVA showed that both diet (F_1,24_ = 71.12, *p* = 0.001) and food concentration (F_2,24_ = 221.10, *p* = 0.001) had significant effects on the CR of *M*. *edulis*; the interaction between both factors (F_2,24_ = 1.44, *p* = 0.256) showed no significant effect (Fig 1a; see S1 File). Similar results were obtained for *M*. *chilensis*, where diet (F_1,24_ = 56.08, *p* = 0.001) and food concentration (F_2,24_ = 7.72, *p* = 0.003) had significant effects on CR (F_2,24_ = 0.36, *p* = 0.700), the interaction between the factors was not significant (Fig 1b; see S1 File). When both bivalves were exposed to different food concentrations of the *A*. *catenella* diet, higher values were observed in *M*. *chilensis*. The two-way ANOVA showed a significant effect of species (F_1,24_ = 94.05, *p* = 0.001) and food concentration (F_2,24_ = 47.14, *p* = 0.001), as well as the interaction between both factors (F_2,24_ = 11.48, *p* = 0.001).

### Exposure to non-toxic dinoflagellate *Alexandrium affine*

**Fig 2:**
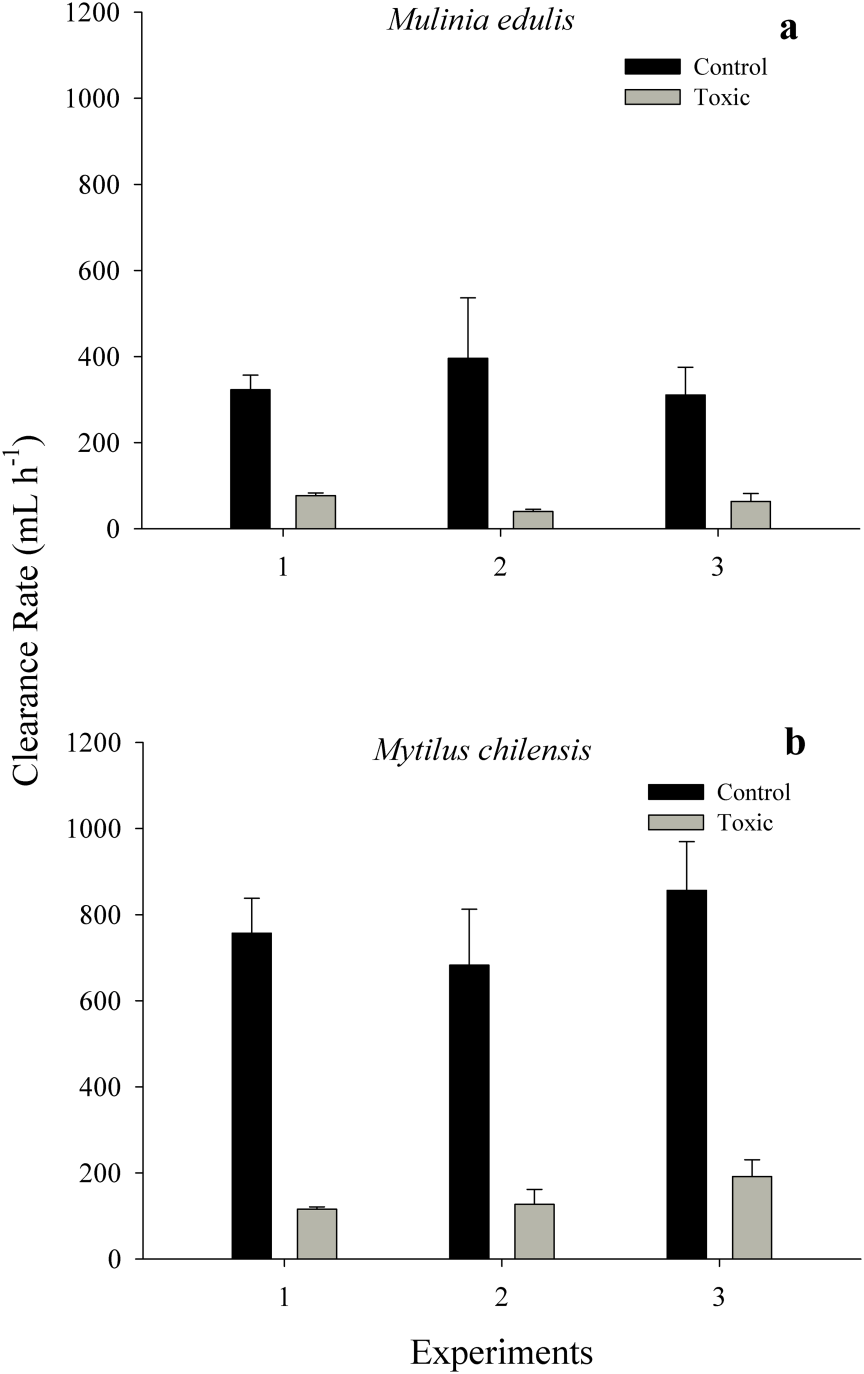
Clearance rate of *Mulinia edulis* (a) and *Mytilus chilensis* (b) exposed to a non-toxic diet (50% *Alexandrium affine*: 50% *Isochrysis galbana*) and a control diet (100% *I*. *galbana*) at 10 mg L^-1^. One experiment was repetead three times. Values represent means ± SE.

The CR of *M*. *edulis* and *M*. *chilensis* fed with the non-toxic *A*. *affine* (10 mg L^-1^) was significantly affected by the factors diet and species (two-way ANOVA; diet: F_1,116_ = 180.87, *p* = 0.001; species: F_1,116_ = 56.67, *p* = 0.001). The interaction between these two factors was not significant (F_1,116_ = 0.001, *p* = 0.978). The CR of *M*. *edulis* fed with the non-toxic *A*. *affine* diet (mean = 60.22 ± 7.06 mL h^-1^) was markedly lower in comparison with the control diet CR (mean = 343.29 ± 49.75 mL h^-1^) (Fig 2a; see S1 File). Similarly, lower values of CR were observed in *M*. *chilensis* when exposed to the non-toxic *A*. *affine* diet (mean = 137.08 ± 15.71 mL h^-1^) in contrast to individuals fed with the control diet (mean = 733.96 ± 62.55 mL h^-1^) (Fig 2b; see S1 File). Note that under both experimental diets (non-toxic *A*. *affine* and control), *M*. *chilensis* showed higher CR values slightly more than double that of *M*. *edulis*.

### Pre-ingestive selection efficiency under a toxic and non-toxic diet

*M*. *edulis* and *M*. *chilensis* exposed at a food concentration of 10 mg L^-1^ have the ability to select particles at the pre-ingestive level, eliminating a larger proportion of the toxic dinoflagellate *A*. *catenella* as well as the non-toxic *A*. *affine* in the form of pseudofaeces. Both bivalves showed differences between the proportions of *I*. *galbana*/*A*. *catenella* and *I*. *galbana*/*A*. *affine* cells in food and pseudofaeces. A two-way ANOVA indicated that pre-ingestive selection efficiency was significant for species (F_1,36_ = 8.92, *p* = 0.005); but not for diet (F_1,36_ = 0.45, *p* = 0.506) nor the interaction of species and diet (F_1,36_ = 3.77, *p* = 0.060). Higher values of selection efficiency were registered in *M*. *edulis* than in *M*. *chilensis* when exposed to the lower food concentration (10 mg L^-1^) of the toxic diet (57.8 ± 7.8% and 33.0 ± 5.6%, respectively). Similar results were observed when these two species were exposed to the diet containing the non-toxic dinoflagellate (*M*. *edulis* mean = 51.5 ± 4.5%; *M*. *chilensis* mean = 46.3 ± 6.0%).

**Fig 3:**
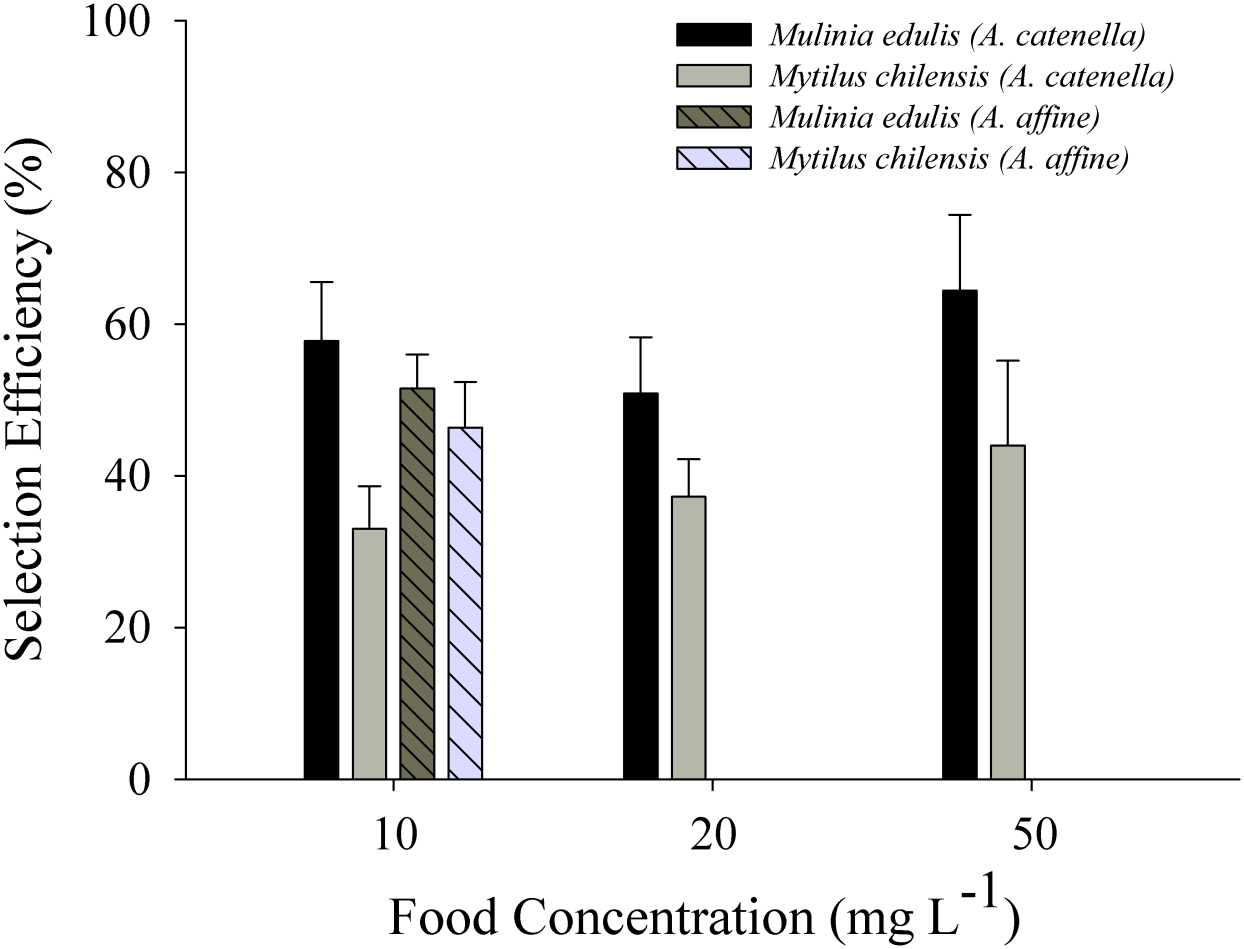
Pre-ingestive selection efficiency of *Mulinia edulis* and *Mytilus chilensis* exposed at three food concentrations (10, 20 and 50 mg L^-1^) containing mixture of 50% *Alexandrium catenella*: 50% *Isochyrsis galbana*. The selection efficiency of *Mulinia edulis* and *Mytilus chilensis* to a non-toxic diet (50% *Alexandrium affine*: 50% *I*. *galbana*) was only measured at 10 mg L^-1^. Values represent means ± SE).

When both species of bivalves were exposed to different food concentrations (10, 20, and 50 mg L^-1^) of the toxic diet (50% *A*. *catenella* + 50% *I*. *galbana*), pre-ingestive selection efficiency was significant for species (F_1,24_ = 94.06, *p* = 0.001), food concentration (F_2,24_ = 47.14, *p* = 0.001), and the interaction of both factors (F_2,24_ = 11.48, *p* = 0.001). Pre-ingestive selection efficiency measured in *M*. *edulis* ranged between 51.5 ± 7.1 and 65.5 ± 10.1% at concentrations of 20 and 50 mg L^-1^, respectively. In *M*. *chilensis*, it ranged between 33.0 ± 6.0 and 44.0 ± 11.0%, at the lower (10 mg L^-1^) and the higher (50 mg L^-1^) food concentration, respectively (Fig 3; see S1 File).

### Endoscopic measurement of particle transport

**Fig 4:**
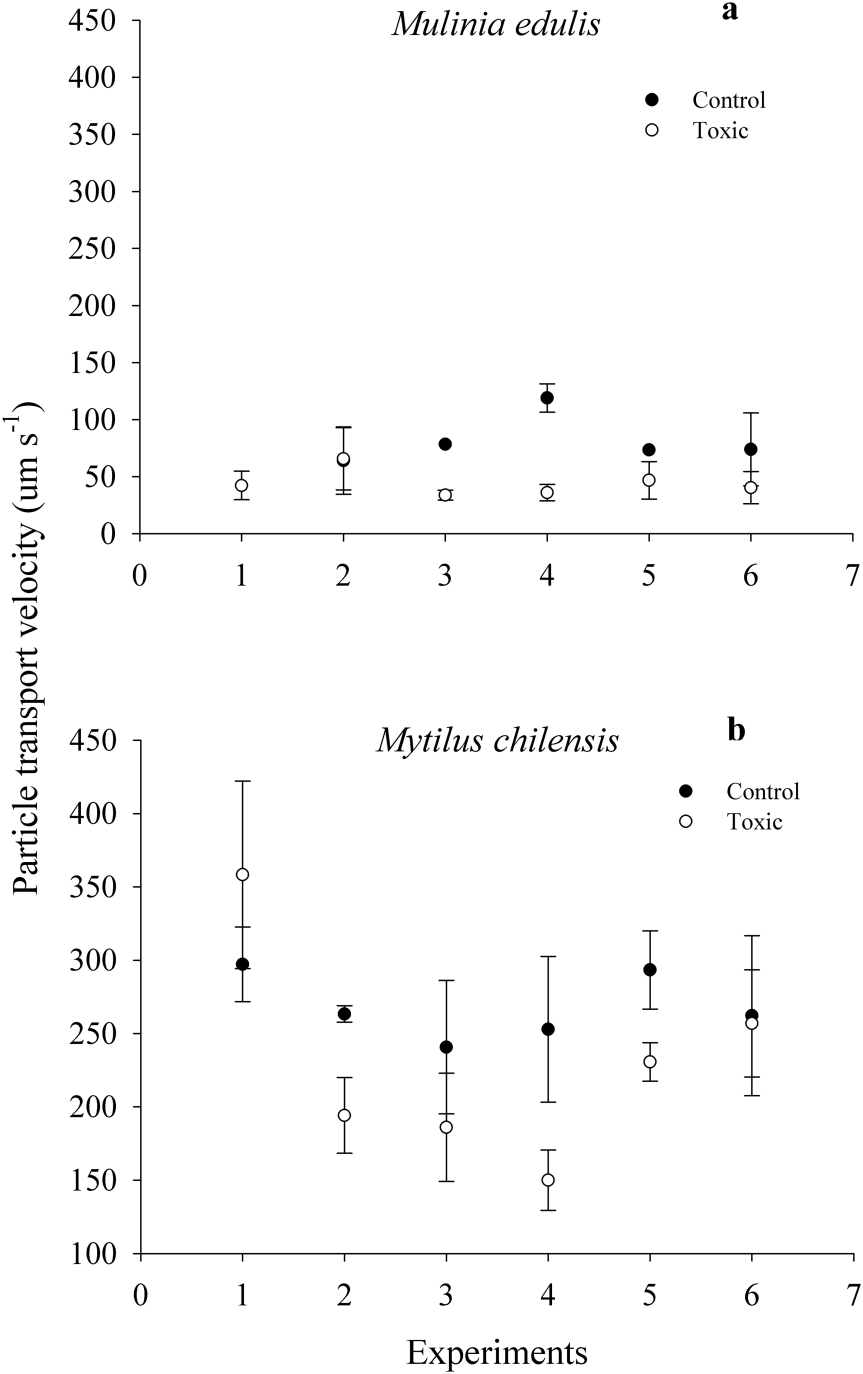
Endoscopic measurements of particle transport velocity (μm s^-1^) in (a) *Mulinia edulis* and (b) *Mytilus chilensis*, during exposure to toxic (50% *Alexandrium catenella*: 50% *Isochyrsis galbana*) and control (100% *I*. *galbana*) diets. Values represent means ± SE.

For *M*. *edulis* particle transport velocity, the one-way ANOVA showed significant differences between diets (F_1,24_ = 5.34, *p* = 0.013). When fed 100% *I*. *galbana* (2.0 mg L^-1^), particle transport velocity had a mean of 82.1 ± 24.6 μm s^-1^; but particle velocity declined to a mean value of 44.5 ± 13.9 μm s^-1^ when fed the toxic diet (50% *A*. *catenella*: 50% *I*. *galbana*) (Fig 4a; see S1 File). In contrast, the one-way ANOVA showed no significant differences in particle transport velocity between diets for *M*. *chilensis* (F_1,24_ = 0.82, *p* = 0.363). Particle transport velocity for the control diet (mean = 268 ± 34.6 μm s^-1^) overlapped with the particle velocity for the toxic diet (mean = 229 ± 32.8 μm/s) (Fig 4b; see S1 File). When results of particle transport rate for both species were included in the analysis, the two-way blocked ANOVA was significant for species (F_1,63_ = 43.851, *p* = 0.001). However, diet (F_1,63_ = 0.459, *p* = 0.501), the block (F_1,5_ = 1.341, *p* = 0.259), and the interaction (F_1,63_ = 0.434, *p* = 0.513) showed no significant effects on particle transport velocity.

## Discussion

The clearance rate of the two bivalve species when fed on the control diet (100% *Isochrysis galbana*) was markedly different and probably reflected adaptations to their respective environments. Navarro et al. [32] found a direct relationship between clearance rate and gill area in four species of bivalves, with the higher clearance rate in the mussel *Mytilus chilensis* being consistent with a larger gill area and living in a less turbid environment. In contrast, the clam *Mulinia edulis* has a smaller gill area and showed a significantly lower clearance rate, typical of infaunal bivalves living in turbid environments [37–39].

The present study provides evidence that the clearance rates of both *M*. *edulis* and *M*. *chilensis* showed a significant reduction when fed a mixed toxic diet of 50% *Alexandrium catenella*: 50% *Isochrysis galba*na. This is comparable to results of earlier studies on the same species [26], in which *M*. *chilensis* had a clearance rate that was reduced 3.6 fold in response to a toxic diet (1.8 mg L^-1^; 50% *A*. *catenella*: 10% *I*. *galbana*: 40% sediment) during its first day of exposure. Widdows et al. [40] observed that *Mytilus edulis*, exposed to the toxic dinoflagellate *Gyrodinium aureolum*, also reduced clearance rates. Shumway and Cucci [14] observed species-specific differences in feeding activity of seven bivalve species in the presence of the toxic dinoflagellate *Protogonyaulax tamarense* strain GT429 (= *Alexandrium tamarense*) and this varied varied with collection locality. Responses included shell valve closure and reduced clearance rates in the clam *Mya arenaria* and the mytilid *Geukensia demissa*. In the same study the bivalves *Modiolus modiolus* and *Spisula solidissima*, were not affected by the presence of the toxic dinoflagellates.

In the present study, *M*. *edulis* and *M*. *chilensis* also showed a decrease in filtration activity as food concentration increased regardless of the diet. This appears to be a generalized behaviour in bivalves, based on measurements collected under natural [7,39,41] and laboratory [42–44] conditions. This inverse relationship between clearance rate and food concentration has also been described for other species of filter-feeding bivalves, such as *Mytilus edulis* [23], *Crassostrea virginica* [45], and *Placopecten magellanicus* [14,46].

When both bivalve species were exposed to a diet (10mg L^-1^) containing the non-toxic dinoflagellate *A*. *affine* (50% *A*. *affine*: 50% *I*. *galbana*), clearance rates decreased by a factor of more than 5 in both *M*. *edulis* and *M*. *chilensis* in comparison to the control diet of 100% *I*. *galbana*. This is larger than the CR reduction induced by the toxic diet (50% *A*. *catenella*: 50% *I*. *galbana)* in *M*. *edulis* (factor of 1.6) and *M*. *chilensis* (factor of 3) in comparison to the control diet (10 mg L^-1^). Although the experiments with *A*. *affine* lasted only 6 hours (due to difficulties in culturing this algal species), previous studies with *A*. *catenella* suggest that this reduction is only temporary, and clearance rate recovery in *M*. *chilensis* was apparent after 1 to 3 days [26,47]. This indicates that both algal diets containing dinoflagellates significantly reduced the CR of both bivalve species. This reduction may be a response to cell size / volume or the shape of the dinoflagellates rather than a direct toxic effect of *A*. *catenella*.

Both *M*. *edulis* and *M*. *chilensis* showed preferential selection for the smaller, non-toxic, *Isochrysis* cells (ca. 5–6 μm), resulting in an enhanced rejection of the larger and toxic, *A*. *catenella* cells (ca. 35 μm) in the pseudofaeces. The higher values of selection efficiency of *M*. *edulis* for diets of both toxic and the non-toxic dinoflagellates can be explained by this species’ larger labial palps [32], and such preferential particle selection has been described for several bivalve species. For example, adults *Crassostrea gigas* demonstrated enhanced rejection of non-toxic *Alexandrium tamarense* in the pseudofaeces [30]. Furthermore, the cockle *Cerastoderma edule* showed selection efficiencies as high as 77% [48], *Mytilus edulis* had selection efficiencies of around 50% [49], and *Cerastoderma edule* from the Exe estuary (southwest England) showed efficiencies between 20 and 60% when exposed to a wide range of particle concentrations (4 to 320 mg L^-1^) [2]. In addition, the effects of the toxic dinoflagellate *Protogonyaulax tamarense* strain GT429 (= *Alexandrium tamarense*) on several species of bivalves demonstrated that *Mytilus edulis* showed no pre-ingestive selection behaviour, with the toxic cells filtered by the gills and subsequently presenting in both the pseudofaeces and the faeces [14]. In contrast, feeding studies with *M*. *arenaria* confirmed preferential rejection of the toxic dinoflagellate in the pseudofaeces and reduced ingestion of toxic cells, resulting in low toxin levels (<50 ug STX 100 g) during the first 10 days [1].

The preferential ingestion of *I*. *galbana* by *M*. *edulis* and *M*. *chilensis* would suggest mechanisms used to reject the two dinoflagellate species at the gills and/or labial palps, presumably based on cell size / shape. Although filter-feeding bivalves have the capacity to ingest particles between 2 and 100 μm [50].

The different clearance rates of the two species were consistent with the observed differences in the transport rate of *I*. *galbana* and *A*. *catenella* cells in the food grooves of the gills. *M*. *chilensis* had a mean transport rate of the toxic diet ca. 3.6-fold higher than *M*. *edulis*, which was the same as the species difference in clearance rates prior to any adaptation to a change in diet. *M*. *chilensis* has previously been shown to have significantly higher particle transport velocities than *M*. *edulis* [32], but that study used a non-toxic diet of pure *I*. *galbana* at a lower particle concentration (ca. 1 mg L^-1^). These results suggest that the lower transport particle velocity observed in the present work is related to the reduced CR, the higher particle concentration, and the presence of larger, toxic dinoflagellate cells. The lower particle transport rate on the gills of *M*. *edulis* reflected the high suspended particle concentrations that the clam experiences in its more turbid environment and the particle sorting and processing that are an adaptation for living in these conditions.

In conclusion, the dinoflagellate species (both toxic and non-toxic species) appear to have inhibitory effects on the feeding / ingestion rate, and therefore growth potential, of both *M*. *edulis* and *M*. *chilensis*, presumably due to the larger algal cells size / shape. In addition, the species differ in their feeding responses to diets, with and without *A*. *catenella* or *A*. *affine*, largely reflecting their adaptations to different environmental conditions. Due to the more turbid waters that characterize their natural environments, the infaunal clam, *M*. *edulis* shows generally lower clearance rates and slower particle transport rates on the gills as an adaptation to cope with higher particle loads. This enables them to sort particles on the gills and palps and achieve higher selection efficiency, which results in a higher rejection of large particles (e.g. sediment and *Alexandrium* spp.). In contrast the mussel, *M*. *chilensis*, is an epifaunal bivalve living in less turbid environments. In Yaldad Bay they are grown from rafts higher in the water column. Consequently, they exhibit a higher clearance rate to increase the collection of food particles from their more dilute environment.

The present findings indicate that the occurrence of dinoflagellate blooms in Yaldad Bay, whether by toxic or non-toxic spp. are likely to have a greater impact on the *Mytilus chilensis* than the infaunal *Mulinia edulis* as a result of their combined effects on clearance rate, selection efficiency and particle transport velocity. The development of HABs produced by the toxic dinoflagellate *A*.*catenella* are occurring with greater frequency and intensity in southern Chile. Therefore the impact of HABs on the functioning of high densities of filter feeding bivalves in the coastal waters and estuaries will have a significant effect on their role in cycling large amounts of particulate matter within the ecosystem.

## Supporting information

S1 File Data for the different physiological variables measured are included in the file S1. (XLSX)
